# Possibility to Use Short Sawn Timber in the Production of Glued Laminated Beams

**DOI:** 10.3390/ma15092992

**Published:** 2022-04-20

**Authors:** Dorota Dziurka, Marcin Kuliński, Adrian Trociński, Radosław Mirski

**Affiliations:** Department of Mechanical Wood Technology, Faculty of Forestry and Wood Technology, Poznań University of Life Sciences, 60-627 Poznań, Poland; marcin.kulinski@up.poznan.pl (M.K.); adrian.trocinski@up.poznan.pl (A.T.); radoslaw.mirski@up.poznan.pl (R.M.)

**Keywords:** structural glulam elements, structural beams, timber, strength properties

## Abstract

Numerous studies have shown that the geometry of micro-joints significantly affects the strength of the so joined timber element. The bending strength increases by creating a larger bonding area by increasing the length of the wedge joint. Although this type of joint has been successfully used for many years, it can still be troublesome to make. For these reasons, the present study investigated an easy-to-fabricate wedge joint, which we folded during the beams’ formation and glued with the same adhesive as the individual lamellas. Although the research has not fully answered all the questions relevant to both scientific and technological curiosity, it indicates the great potential of this solution. Following the principle adopted in the ongoing wood optimisation work, we concluded that the beams of the target cross-section should be produced, and it should only be possible to cut them to a certain length. In this approach, we only removed defects at critical points for the beam structure and, in this way, up to 30% of the timber processed could be saved or better utilised.

## 1. Introduction

The growing demand for wood-based materials has led to an increased demand for timber, resulting in the accelerating competition between individual branches of the wood industry for raw material resources. In contrast to the common practice in the 20th century, currently, the plywood and veneer sectors use sawn timber, and the sawmilling industry increasingly processes timber of smaller diameters and material harvested from much younger age classes, while the board manufacturing industry, except for OSB (Oriented Strand Board) producers, has practically ceased to purchase roundwood. In Poland, the particleboard industry no longer utilises timber grade S2a, i.e., medium-sized timber measured in stacks of rollers of 120–200 cm in length and a top log diameter of a min. of 5 cm. This transition to smaller sized timber is related to the use of much younger wood, in which the proportion of so-called juvenile wood increases. Considerably worse physico-mechanical properties characterise such wood than mature wood [1]. In many countries, plantations of fast-growing trees are established to meet the increasing demand for timber [2]. In this case, the share of juvenile wood is even greater [3]. In Poland, it is assumed for pine wood that juvenile wood is found up to the 16th annual growth ring, and it should be avoided in the manufacturing of structural elements [4]. However, irrespective of the degree of its maturity, wood is characterised by an extensive range of physico-mechanical properties within a single species between individual specimens and a given specimen [5]. Conversion of long timber produces sawn timber exhibiting an extensive range of mechanical properties, particularly bending strength or tensile strength, as well as the modulus of elasticity. Defects revealed on the surface determine a technical quality of a given timber specimen. A solution facilitating a more advantageous use of timber in building structures is manufacturing glued laminated timber, i.e., glulam. Its layered structure makes it possible to arrange individual lamellas depending on their technical quality [6,7,8,9].

Glulam beams are typically bonded using MUF (Melamine-Urea-Formaldehyde) resin or other synthetic compounds [10,11,12]. Solutions have also been developed to apply environmentally-friendly adhesives [13]. Nevertheless, the implementation process for new adhesives will be gradual, as the applied glues have to ensure good bonding between individual lamellas and bonds needed to elongate individual lamellas. In the case of structural timber, finger joints are common. Many studies have tested and investigated these joints as applicable for bonding structural timber and in the furniture industry [14,15,16,17,18]. Various authors indicate different ranges of joint length from 6 mm to 25 mm, which may be used in structural timber [19,20,21]. However, it results from the investigations conducted by the authors of this study that short joints, typically used in furniture production, carry greater loads than those for the primary beam grade, GL24. These guidelines show that this strength needs to be at least approx. 40% greater than the assumed beam strength, whereas it may be attained very rarely.

It results from numerous studies by Rapp [22,23,24,25,26] and Özçiftçi and Yapıcı [27] that the geometry of finger joints has a significant effect on the strength properties of such bonded wooden elements. Additionally, the bending strength grows with the increase in the bonded area thanks to the elongation of the finger joint. Although such joints have been successfully used for many years, they may prove difficult to execute. As previously mentioned, the required quality of a given lamella in a glulam beam structure depends on its position at the beam cross-section.

Because of the above, it was decided, in this study, to analyse wedge joints, which are easy to manufacture and are assembled during beam formation and resinated with the same adhesive as individual lamellas.

## 2. Materials and Methods

A rational approach to glulam beam manufacturing is to comprehensively analyse the quality of obtained sawn timber and its bucking. The latter aims to lay in the individual zones of the beam only that piece which, in the designed arrangement, can transfer the resulting stresses. Thus, it may be necessary to remove a defect, which will result in the formation of a too-short beam. Generally, sawn timber may be bonded end to end using finger joints. However, a technological line producing the finger joints’ purchase cost exceeds 5 to 6 times the press price for beam bonding.

**Figure 1:**
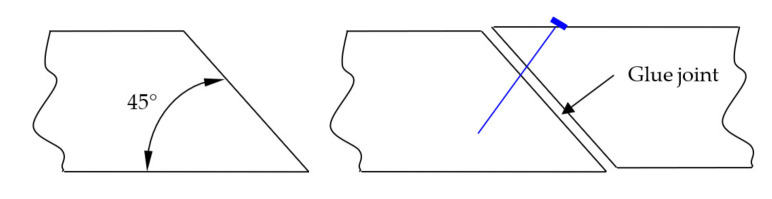
The binding system proposed in this study.

For this reason, most small sawmills in Poland do not buy such machines but instead try to buck available timber material to produce lamellas of the required length. It seems that a simple solution to this problem may be provided by simplifying the joint finger system, explicitly by applying a wedge joint with a glue line. For this reason, it was decided to analyse such a solution using a joint made at an angle of 45° (Figure 1). In this experiment, the formed surfaces were bonded with the same adhesive as the one used to bond beams during the formation of beam sets. Since layers coated with the adhesive in the course of their formation in the press tend to shift, it was decided to nail the bonded fragments using a pneumatic nailer with four nails driven at approx. 25 mm spacing. The nails were placed at a certain angle to prevent binding the glued elements with the sawn timber below them.

**Figure 2:**
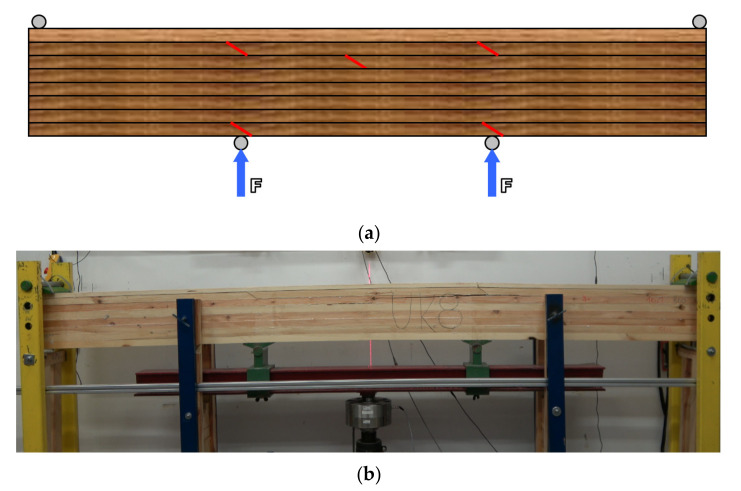
Measuring device: (**a**) the scheme of the manufactured beam, (**b**) photo.

It was decided to manufacture beams composed of 8 layers of the primary yield (variant M) or 7 layers of the primary yield and one sideboard (variant UK). Generally, this change aimed to determine the effect of face thickness covering the wedge-jointed specimens simultaneously. It was assumed that such a joint might be used only in the deeper layers rather than the face layer exposed to tensile forces. Thus, the designed beams had a face lamella of 38 mm in thickness (variant M) and 22 mm in thickness (variant UK). The manufactured set comprised three end-to-end bonded timber specimens (Figure 2). The end-to-end bonded timber was found in the second, third, and eighth lamellas, counting from the tensile zone.

Moreover, lamellas nos. 2 and 8 had two wedge joints, while lamella no. 3 had only one. In the compressed zone, the joints were located in the vicinity of the pressure points.

Beams were manufactured according to standard practice. An adhesive MUF 1247 at 220–240 g/m^2^ mixed with a curing agent at 10% to MUF dry matter was applied onto the sawn timber surface. The process of press charging lasted up to 20–22 min. After the beams were fed into the press, it was closed, and the assumed pressing pressure of approx. 0.48–0.50 MPa was applied for a min. of 4 h. Pressing was conducted with the use of an industrial press equipped with hydraulic cylinders dedicated to the production of glued structural elements (FOST, Czersk, Poland). The beams were manufactured based on EN-14080 [9], with modifications according to the purpose of the study. For each type, eight beams were manufactured. After conditioning, the beams were tested to determine their strength and the modulus of elasticity in a 4-point bending test. The choice of the test was based on the assumptions of the EN 408 standard [28]. It was equipped with: a hydraulic cylinder (50 Mg, Hi-Force, Daventry, UK), hydraulic pump (50 Mg, Hi-Force, Daventry, UK), oil flow rate regulator (Hi-Force, Daventry, UK), force sensor (CL 16 tm 500 kN, ZEPWN, Marki, Poland), and deformation sensor (KTC-600-P, Variohm Eurosensor, Towcester, UK). The moisture content of each piece was determined using a Tanel HIT-1 hammer moisture meter.

To determine the effect of the joint quality itself on the strength of the tested elements, the tensile strength of the specimens, prepared in the same way as in the manufacturing of the beams, was determined. For this purpose, pine lumber, 40 mm thick, was cut perpendicularly to the fibers at an angle of 45°. The resulting two fragments were glued together. MUF, vinyl dispersion, and polyurethane adhesives were used for gluing. After gluing, specimens of approximately 20 cm in length and with a 20 mm × 20 mm cross-section were cut. The specimens were then evaluated for tensile strength along the fibers. As expected, the adhesive bond failed, so the results should have been related to its strength. The results of the experimental measurements were analyzed using a STATISTICA 13.0 package (Version 13.0, StatSoft Inc., Tulsa, OK, USA).

## 3. Results and Discussion

The beams were manufactured from sawn timber subjected to an assessment of the modulus of elasticity in a 4-point bending test, as reported in the earlier publications [7,8]. Table 1 shows the average values of the modulus of elasticity of the individual lamellae. A very high modulus of elasticity characterised the lumber used in the study. It is assumed that the average modulus of pine lumber is between 11 GPa and less than 12 GPa. The need to use lumber with a high modulus of elasticity resulted from the availability of lumber and the previous research [7].

In our study, except for the first lamella, the quality of the lumber, determined by the modulus of elasticity in the remaining layers, was very similar, and the differences did not exceed 5%. However, the first layer differed quite significantly because, in this case, M-type beams were produced from thicker lumber (38 mm) and with a higher modulus of elasticity by over 3 GPa. Therefore, this was a reduction of the modulus of elasticity of almost 20%, which could also be important when assessing the bending strength. According to EN 338 [29], the lumber used would be assigned to classes C35–C50. Based on the research of Steiger and Arnold [30], we could assume that the modulus of elasticity evaluated in the bending test would be equal to that which we determined in the tension test.

**Figure 3:**
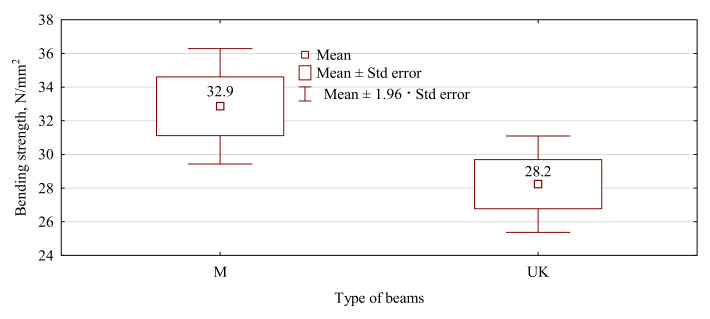
Static bending strength in a 4-point test for manufactured beams.

**Figure 4:**
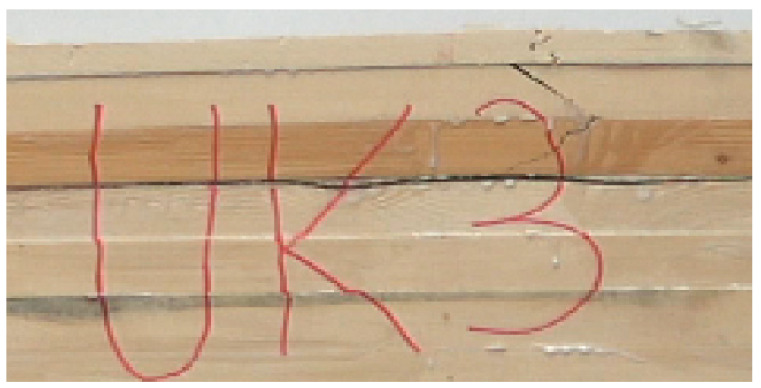
Failure of joint in beam-type UK.

The strength of such manufactured beams was much lower than that of the beams produced within a similar standard [7]. In the earlier studies, the bending strength of beams manufactured in terms of the modulus of elasticity amounted to approx. 40–44 N/mm^2^, while in this case, it was lower and, on average, 30 N/mm^2^ (Figure 3). Thus, it was equivalent to a reduction of strength by almost 30%. However, the strength of such designed beams continues to be high and is much higher than 24 N/mm^2^. The lowest strength recorded for the tested specimens was 24.05 N/mm^2^, which means both batches could be classified as grade GL24c, which is the basic grade for structural beams. However, in our study and as it results from the values presented in Figure 3, the strength of the beams manufactured using sawn timber of 22 mm in thickness was approx. 4.5 N/mm^2^ lower than the beams with faces composed of sawn timber of 38 mm in thickness. To statistically compare the results for both batches, an analysis of variance and a Student’s t-test were performed. The study of the variance (Table 2) showed no grounds for the rejection of the hypothesis stipulating the equality of the variance for both batches. Interestingly, there were also no grounds for a statement of the strength of beams manufactured with a thinner face layer (22 mm) being statistically significantly worse than beams with a face of 38 mm in thickness. Nevertheless, the value of this statistic is relatively low with the assumed a = 0.05, and it may be expected that in a larger sample, the differences could be more significant. What is essential, in the case of the type M beams in both cases, although this is as much as 25% of the tested beams, is that a simultaneous failure of the joint was observed at beam failure. While the cause of the failure was not definitely identified, the significant effect on the joint should not be excluded, as it is essential since the wedge joint failure was recorded in the tensile zone (Figure 4).

**Figure 5:**
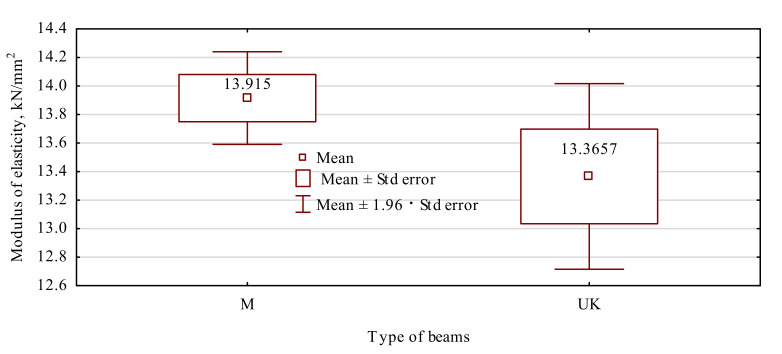
Modulus of elasticity of manufactured beams.

The mean modulus of elasticity for the beams manufactured in this experiment was relatively high and amounted to over 13 GPa, while the lowest value was much higher than 11.2 GPa (Figure 5). In this case, such beams may be classified as minimum grade GL 30c. However, as mentioned earlier, in terms of bending strength, the manufactured beams may be classified as grade GL24c. The stiffness of the beams is thus much greater than their potential to carry loads. In our study, up to the load amounting to around 30% of apparent strength, no effect on the beam joint’s manner was manifested. The conducted analysis of the variance indicates (Table 3) that a comparable value of variance characterises both variants and that there are no grounds for rejecting the zero hypothesis on the equality of the variance. Thus, the Student’s t-test needs to be considered as appropriately conducted. This analysis indicates that the moduli of elasticity for both batches/types of beams are similar.

**Figure 6:**
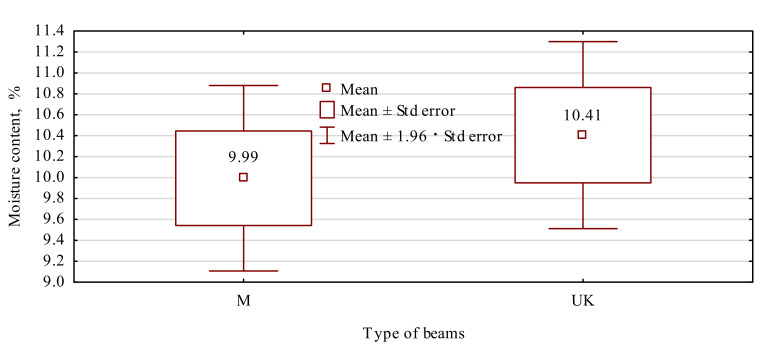
Moisture content of manufactured beam types.

Similarly, as was in the case of the static bending strength and modulus of elasticity, statistical analyses were conducted to assess the beam moisture content during tests of their mechanical properties. The results of these analyses are given in Table 4 and Figure 6. In our study, the moisture content of both batches fell within the range of the moisture contents of beams for indoor use and amounted to 10% and 10.4% for beams type M and UK, respectively. As the statistical analysis resulted, both beam batches were tested at similar moisture contents. Thus, the results of both samples may be compared even without the conversion of the results into characteristic moisture content.

When beams are subjected to the bending test, they develop normal stresses in their structure, which are essentially related to three quantities, i.e., bending moment, a moment of inertia, and the distance of the fibers/cross-sections from the neutral axis. Table 5 shows the values of normal stresses acting on individual lamellas, depending on the distance from the neutral axis. In our study, the values of the force acting on the bale were taken as the strength values, allowing them to obtain, for the given cross-sections, the strength corresponding to the C35 lumber class for the UK-type beams and C35 and C50 for the M-type beams. It turns out that M-type beams carry a slightly lower load than predicted for class C35; the values are lower by about 6%. Alternatively, it is as much as about 20% for UK beams.

**Figure 7:**
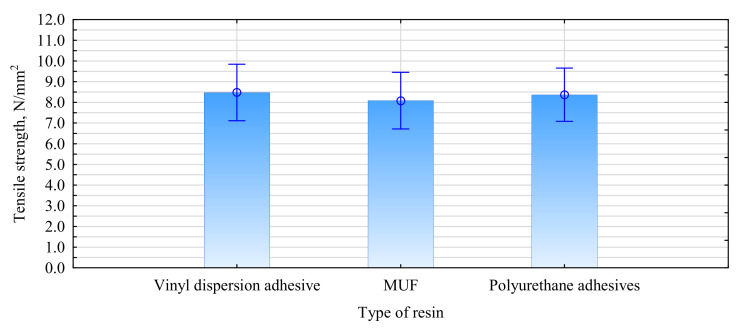
ANOVA analysis of the tensile strength of adhesive joints.

**Figure 8:**
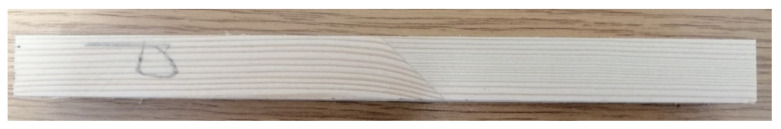
View of the tensile specimen (cross-section approx. 20 × 20 mm).

No shearing of the glue joint was observed during the bending test, and the results show that its strength is relatively high at 8 N/mm^2^ (Figure 7). We evaluated this in a tensile test of specimens with the glue joint positioned at 45°, as was done in the beams (Figure 8). In addition, we evaluated two other adhesives commonly used in wood-working with resistance class D3. It turned out that all adhesives showed a similar joint tensile strength.

Consequently, the results show that it is the weakest element of the piece, but many more factors determine its failure than the value of normal stress alone. The influence of the moduli of elasticity of the individual lamellas and of the adhesive resin itself must also be considered [31,32].

## 4. Concluding Remarks

It seems that the conducted experiments, while not fully answering all the important questions from the scientific and technological point of view, indicate a considerable potential of this solution. It remains an open issue whether each beam design (the relation of its height to the thickness of the face layer) will make it possible to use this type of solution. In the adopted beam configuration, the second lamella in type UK carries stresses as much as 45% greater than the second lamella in type M.

These dependencies may be even greater in the 20/40/40/40/40/20 system (lamella thickness), in which the second lamella transfers stresses as much as 75% greater than the second lamella in the 40/40/40/40/40/40 beam. The quality of the manufactured beams was sufficient to meet the basic strength class of the glued laminated beams, i.e., GL24c.

According to the principle adopted within the conducted tests for optimising the use of the wood, beams with a target cross-section should be produced, and it should only be possible to cut them to a certain length.

## Figures and Tables

**Table 1:** Quality of lumber used in the research and determined by the measurement of the modulus of elasticity.

Type of Beam	Characteristic	Number of Lamella (Counts from the Tension Zone)							
		Lam. 1	Lam. 2	Lam. 3	Lam. 4	Lam. 5	Lam. 6	Lam. 7	Lam. 8
M	E_m_ (kN/mm^2^)	16.36	15.51	15.48	17.73	17.26	17.21	17.65	15.41
	SD (kN/mm^2^)	0.30	0.44	0.33	0.14	0.18	0.20	0.18	0.31
	v (%)	1.86	2.85	2.14	0.79	1.04	1.14	1.02	2.04
UK	E_m_ (kN/mm^2^)	13.24	16.08	16.15	17.63	17.18	17.21	17.66	15.73
	SD (kN/mm^2^)	0.49	0.78	0.44	0.13	0.12	0.15	0.14	0.49
	v (%)	3.72	4.88	2.73	0.74	0.70	0.89	0.80	3.11
Δ	E_mM_ − E_mUK_	3.13	−0.56	−0.67	0.10	0.09	0.00	−0.01	−0.31

M—beams composed of 8 layers of the primary yield; UK—beams composed of 7 layers of the primary yield and one sideboard; E_m_—modulus of elasticity; SD—standard of deviation; v—coefficient of variation.

**Table 2:** Statistical analysis of bending strength.

t *	df **	p ***	Standard Deviation M	Standard Deviation UK	Levene’s F(1,df)	Levene’s df	Levene’s p
1.999	13	0.066956	4.949	3.861	0.164888	13	0.691301

* t—value of the Student’s *t*-test, ** df—the number of degrees of freedom, *** p—Probability value.

**Table 3:** Student’s *t*-test for the modulus of elasticity, Levene’s analysis of variance.

t	df	p	Standard Deviation M	Standard Deviation UK	Levene’s F(1,df)	Levene’s df	Levene’s p
1.54087	13	0.14733	0.46856	0.87850	1.7212	13	0.21224

**Table 4:** Statistical analysis of the moisture content of beams.

t	df	p	Standard Deviation M	Standard Deviation UK	Levene’s F(1,df)	Levene’s df	Levene’s p
−0.63937	13	0.53369	1.2782	1.2051	0.58921	13	0.456442

**Table 5:** The values of normal stresses acting on individual lamellas.

Force F (kN)	M_g_max_ (kN·m)	Beam Type	Lam_1		Lam_2		Lam_3		
			Top *	Axis **	Top	Axis	Top	Axis	Bottom ***
			Normal Stresses (N/mm^2^)						
121	67.2	UK	35.0	32.3	29.7	25.1	20.5	15.9	11.3
134	74.4	M	35.0	30.6	26.3	21.9	17.5	13.1	8.8
191.5	106.3	M	50.0	43.8	37.5	31.3	25.0	18.8	12.5

*—upper surface of the lamella, **—in the axis of the lamella, ***—lower surface of the lamella.

## Data Availability

The data presented in this study are available on request from the corresponding author.
